# Characterization of Endothelial Progenitor Cell: Past, Present, and Future

**DOI:** 10.3390/ijms23147697

**Published:** 2022-07-12

**Authors:** Amankeldi A. Salybekov, Shuzo Kobayashi, Takayuki Asahara

**Affiliations:** 1 Kidney Disease and Transplant Center, Shonan Kamakura General Hospital, Kamakura 2478533, Japan; shuzo@shonankamakura.or.jp; 2 Shonan Research Institute of Innovative Medicine, Shonan Kamakura General Hospital, Kamakura 2478533, Japan; 3 Qazaq Institute of Innovative Medicine, Nur-Sultan 010000, Kazakhstan

**Keywords:** endothelial progenitor cells, endothelial colony-forming cells, hemogenic angioblasts, regeneration-associated cells, resident endothelial progenitor cells, extracellular vesicles

## Abstract

Endothelial progenitor cells (EPCs) are currently being studied as candidate cell sources for revascularization strategies. Despite these promising results, widespread clinical acceptance of EPCs for clinical therapies remains hampered by several challenges. The challenges and issues surrounding the use of EPCs and the current paradigm being developed to improve the harvest efficiency and functionality of EPCs for application in regenerative medicine are discussed. It has been observed that controversies have emerged regarding the isolation techniques and classification and origin of EPCs. This manuscript attempts to highlight the concept of EPCs in a sequential manner, from the initial discovery to the present (origin, sources of EPCs, isolation, and identification techniques). Human and murine EPC marker diversity is also discussed. Additionally, this manuscript is aimed at summarizing our current and future prospects regarding the crosstalk of EPCs with the biology of hematopoietic cells and culture techniques in the context of regeneration-associated cells (RACs).

## 1. Introduction

The discovery of endothelial progenitor cells (EPCs) has shed light on the existence of postnatal vasculogenesis in humans [1,2]. Since the discovery of EPCs, many research studies have been accumulated regarding the biology of EPCs, culture assays, surface markers, origin, and differentiation hierarchy, including translational animal studies and further clinical applications. However, this field has accumulated unresolved issues such as ambiguous or controversial findings with regard to EPC origin and biological characteristics. This has facilitated the unification of EPC-related terminology based on the progress of recent research because of the critical role of EPCs in vascular repair in health and disease, and, in some cases, progress toward their use in cell therapy seemed to be a proper step forward in this area [3]. This manuscript summarizes the current understanding of in vitro and in vivo EPC characterization, differentiation, origin, and the crosstalk between hematopoietic cells.

## 2. From Hemogenic Angioblasts to EPCs

Hematopoietic stem cells (HSCs) in adults are believed to be derived from hemogenic endothelial cells (HECs) in mid-gestational embryos [4]. Particularly, hematopoietic stem and progenitor cells (HSPCs) are visualized to emerge from aortic endothelial cells (ECs), also known as the embryonic aorta-gonad-mesonephros area, via a transient and dynamic process called the endothelial-to-hematopoietic transition to form intra-aortic hematopoietic clusters [5,6,7]. Being located within intra-aortic hematopoietic clusters or deeper subendothelial layers, preHSCs serve as important cellular intermediates between HECs and HSCs, featured by their inducible repopulating capacity and priming with hematopoietic surface markers [5,6]. Subsequently, based on the current concept of de novo generation of HSPC from the precursor of hemogenic endothelial cells, namely hemogenic angioblasts, the latter is thought to migrate to and propagate within the fetal liver and fetal bone marrow as those tissues develop [4,5,7]. A previous mouse study identified that adult bone marrow-derived, phenotypically defined hematopoietic stem cells (c-kit+, Sca-1+, lineage−) give rise to functional endothelial cells [8]. Later, Case et al. isolated CD34+ CD45−/+ cells and, even further, stringently selected for downstream EPCs markers such as CD34+ AC133+ VEGFR-2+ and assayed for either EPC or HPC colony-forming assay [9]. Notably, CD45+ CD34+ CD34+ AC133+ VEGFR-2+ cells formed an HPC colony but not EPCs, while CD45− CD34+ CD34+ AC133+ VEGFR-2+ cells formed classical endothelial cell-like morphology, had highly proliferative potential, and expressed the CD31 marker [10]. More recently, Ratajczak et al. described the successful isolation of very small embryonic-like stem cells (VSELs) from umbilical cord blood. Morphologically, VSELs are small cells, corresponding in size to cells in the inner cell mass of a blastocyst, and, depending on measurement conditions (in suspension or after adhesion to slides), they measure ≈3 to 5 μm in mice and ≈5 to 7 μm in humans. The authors defined that VSELs are at the top of the stem cell hierarchy in normal bone marrow, with negative expression of the CD45 marker, giving rise to HSCs, mesenchymal stem cells, and EPCs [9].

Taken together, the accumulating results suggest that hemogenic angioblasts can give rise to HSCs and EPCs. It seems that either peripheral blood or umbilical cord blood-derived CD34+ CD45− cells are mainly composed of hemangioblasts, and their number and proliferative or self-renewal capacity reduce in the elderly population.

## 3. EPC Characterization Ex Vivo: From Past to Present

**Figure 1:**
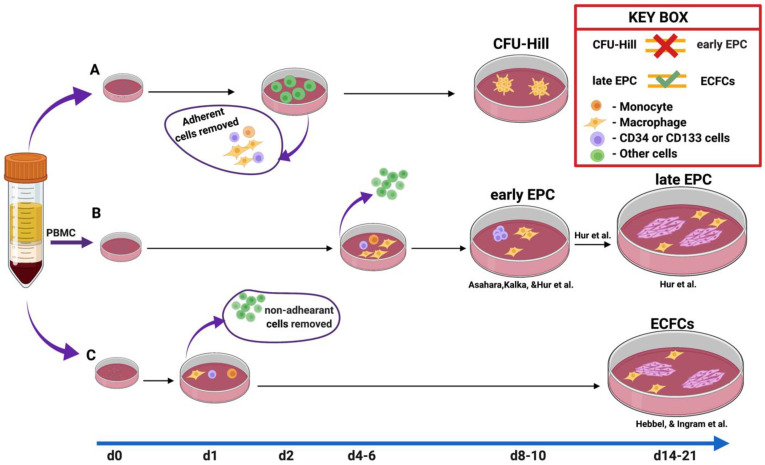
The methods of EPC culture. Method A is the colony-forming units of Hill. In this method, the adherent cells were depleted at day 2/48 h due to the author’s concern regarding contamination by “circulating endothelial cells”. Nonadherent cells were plated to the dish to count CFU-EC or CFU-Hill from days 5 to 9. Method B was developed to enhance EPC ex vivo. The nonadherent cells were discarded on days 4–6, while adherent cells were further incubated up to day 10 in the studies of Kalka [11] and Asahara et al. [1] Subsequently, Hur et al. [16] used the same method as Kalka et al., [11] with slight modifications such as the prolongation of incubation time. Notably, they observed endothelial outgrowth cells at day 14 in the midst of early EPC, which indicated that some of the hidden cells among an early EPC population could give rise to ECFCs. Thus, CD34+ and CD133+ cells depleted mononuclear cells could not form ECFC, whereas the presence of these cells successfully did. In the last C method, initially described by Hebbel et al. [22] and later modified by Ingram et al., 1 day after plating the cells, nonadherent cells were removed. At days 14–21, the blood endothelial outgrowth cells appeared morphologically and phenotypically similar to late EPC.

The term “EPC” has been grouped into several distinct cell subtypes since the first original findings, and some of them cannot be characterized as EPC due to their divergent biological function, contribution to neoangiogenesis, lack of distinct delineating immunophenotype markers from other cell lineages, and origin. To this end, several groups of laboratories have attempted to summarize all EPC-related terms to have a consensus statement on the endothelial progenitor nomenclature [3]. Notably, the consensus statement distinguishes proangiogenic cells from “true EPCs” for our better understanding; on the other hand, the statement does not cover whole terminologies that have been utilized in the scientific literature so far. Thus, we have returned back to the original introduction of EPCs and development stages along with endothelial outgrowth cells and tissue-resident EPCs to group them appropriately and define markers to delineate EPCs from other cells. Initially, EPCs were characterized as CD34, KDR, and VE-cadherin positive cells that were purified by magnetic beads. When these cells were plated on a fibronectin-coated plate, they attached and expressed eNOS, Flk-1/KDR, and CD31 and finally released NO, indicating a full set of endothelial cell characteristics [1]. Taking into account that EPCs are extremely rare in circulating blood, to generate a sufficient number of cells, researchers have developed an ex vivo expansion culture system to expand rare EPCs using whole blood peripheral mononuclear cells (PBMCs). Kalka et al. introduced an ex vivo EPC expansion method using several growth factor cocktails [11]. The PBMCs were plated on culture dishes coated with human fibronectin for *4 days,* and then *nonadherent cells were discarded*, and *adherent* or *attached cells* were maintained by replacing the media in the culture for up to 10 days (Figure 1B). This culture technique facilitated Hill et al.’s [12] group to develop a new growth media combination and to evaluate the endothelial cells colony-forming unit (CFU-EC or also known as CFU-Hill) in vitro with significantly different protocols by utilizing PBMCs. The difference between CFU-Hill vs. Kalka is that PBMCs were resuspended in an endothelial growth medium and plated on fibronectin-coated dishes for *48 h* to avoid contamination by “*circulating endothelial cells*”; subsequently, *nonadherent* cells were seeded to another fibronectin-coated dish for 7 days to count the colonies (Figure 1A). Due to ease of use, CFU-Hill produced a commercial kit to evaluate the EPC number, which was highly correlated with the Framingham cardiovascular risk factor score. However, several laboratories were concerned that these colonies were not “EPCs” and, rather, were mainly composed of myeloid and lymphoid cell subsets that were unable to form blood vessels in in vivo experiments [13,14,15]. Subsequently, Hur et al. [16] identified two types of EPCs, namely early and late, by applying or modifying the Kalka et al. method as previously mentioned [11]. The first media replacement occurred at *day 6*; thereafter, the media were changed every 3 days until late EPC appearance. Notably, after 2 weeks of plating, they observed distinct morphology colonies from early EPC, so-called late EPC, or ECFC in the midst of early EPC (Figure 1B,C). The early EPCs secrete more angiogenic cytokines or paracrine factors, as the majority of them are composed of “circulating angiogenic cells,” including monocyte/macrophages and lymphocytes, while late EPCs appeared 2 to 3 weeks after plating with a “cobblestone” morphology. In an animal study, both types of EPCs equally returned perfusion of the hindlimb ischemia (HLI) to levels that were similar to those recorded in the contralateral nonischemic hindlimb. Undoubtedly, the bona fide EPCs derived from the rare heterogeneous CD34+ cells were hidden in early EPC populations [16,17,18]. Over time, several controversial findings have accumulated in this field. Some authors have claimed that CD34 or CD133 is not an EPC marker [17], while other authors improperly or equally grouped early EPC and CFU-Hill culture techniques as the same method [3,18] (Figure 1, see Key Box). Previous studies have compared cord blood and PB-derived CD34+ cells in the presence or absence of hematopoietic CD45 surface antigen expression between CFU-Hill vs. late EPCs, but not in the classical early EPC culture technique. This experiment concluded that CD34+ CD45− cells generated late EPC, while the CD34+ CD45+ fraction did not; obviously, CFU-Hill may not form late EPC if attached cells were removed 48 h after plating. Moreover, compelling evidence suggests that there is crosstalk between myeloid and lymphoid cells and EPC, which may exhibit EPC expansion [19]. Notably, our group and others have demonstrated that CD34 depleted either CB or PBMCs unable to generate any late EPC colonies or ECFCs, suggesting that late EPCs are derived from CD34+ cells [20,21].

## 4. Ex Vivo Culture Environment May Change the EPC Surface Marker

Recently, Rossi et al. found the intracellular expression of the CD133+ antigen marker in ECFCs [23]. When they transplanted the CD133 molecule silenced ECFCs into HLI-induced mice, revascularization was significantly abolished, whilst the ECFCs transplantation was recovered, indicating that intracellular CD133 antigen expression is crucial for ECFC maintenance [23]. Another report demonstrated that during cell culture, CD34− expressed ECFCs re-expressed CD34+. In addition, cell–cell contact formation and serum supplements significantly modulated the CD34 expressional profile. Functionally, these two populations are not identical: CD34+ ECFCs exhibited a higher tube-forming capacity and tip cell gene expression compared with CD34− cells [24]. A previous study depicted the existence of a population of dormant human HSCs that are CD34− but become positive (CD34+) just prior to cell division [25]. The CD34+ cell is not just a marker; rather, it plays an essential role, for it is in the first prerequisite step of cell migration via vascular selectin binding [26]. The latest position paper on ECFCs encourages the use of 10% FBS instead of 5%; the latter significantly influences ECFC colony formation ability [27]. The latest research studies on EPC have also raised awareness of EPCs culturing on fibronectin or collagen types or on using fetal bovine serum; the latter releases extracellular vesicle cargo containing growth factors, mRNA, or miRNA, which may facilitate the proliferation and differentiation of cells, while their concentration is higher in the culture medium [28,29,30,31,32]. Another group was also concerned regarding the functioning of such cells cultured endothelial CFU in vivo, similar to the circulating cells. Thus, even single-cell cultures with strict selection may not be excluded from the probability of “*reprogramming*” in vitro by an artificial milieu, which was reported formerly [18]. Taking into account the above issues, we have developed a clonogenic assay to address the primary circulating EPCs from different origins: peripheral blood, bone marrow, and cord blood, respectively. From the single cord blood-derived CD133+ cell, two types of colonies were raised at day 18 after plating into a semisolid culture dish, termed as small and large EPC-CFU. The large EPC-CFU was observed to highly express Ve-cadherin, vWF, etc., and express CD45 and CD14 markers less in comparison with small EPC-CFU. Notably, the single-cell culture results indicated the presence of populations that can give rise to both endothelial and hematopoietic progenitors in vitro at a ratio of 24% without VEGF or 36% with VEGF, indicating that the growth factor may “*reprogram*” the cells. The hemato-endothelial commitment assay disclosed that large EPCs gained endothelial commitment and differentiation. Moreover, the transplantation of large EPCs markedly amplified blood flow perfusion in the HLI model by enhancement of the neovasculogenesis process [20].

### Classical EPC Surface Marker Characteristics

**CD34** is a 110-kDa transmembrane glycoprotein present on leukemic cells, EC, and stem cells. In addition, it is localized on cells of the splenic marginal zone, dendritic interstitial cells around vessels, nerves, hair follicles, muscle bundles, and sweat glands in a variety of tissues and organs [26,33].

**CD31**, a member of the immunoglobulin superfamily, is a 130-kDa transmembrane glycoprotein also designated as platelet endothelial cell adhesion molecule 1. It is present on the surface of platelets, monocytes, macrophages, and neutrophils and is a constituent of the endothelial intercellular junction [33]. It plays a major role in the adhesion cascade between EC and inflammatory cells during inflammation in facilitating leukocyte migration and between EC during angiogenesis.

**vWF** (factor VIII-related antigen) is a glycoprotein that mediates platelet adhesion to subendothelium at sites of vascular injury and binds and stabilizes factor VIII in the circulation. It appears to be expressed exclusively on EC, where it shows a granular pattern of reactivity. It is also expressed in the cytoplasm of megakaryocytes. It is stored in Weibel–Palade bodies [33,34].

**CD146**, a 113-kDa glycoprotein in the plasma membrane, plays a key role in the control of several vessel functions. Three forms of CD146 have been described, including two transmembrane isoforms and a soluble protein that is detectable in the plasma. These CD146 forms mediate pleiotropic functions through homophilic and heterophilic interactions with proteins present on surrounding partners. The cytoplasmic regions of the long CD146 and short CD146 isoforms exhibit strong homologies between human and mouse, at 93% and 95% similarity, respectively. The short isoform of CD146 is generated through the shedding of the extracellular portion of long CD146 and short CD146 isoforms, and it exhibits a molecular weight of 100 kDa compared with ≈113 kDa for membrane isoforms [35].

**CD144**, also known as VE-cadherin (vascular endothelial cadherin), is a type of cadherin, and it is encoded by the human gene *CDH5.* VE-cadherin is of vital importance for the maintenance and control of endothelial cell contacts. Mechanisms that regulate VE-cadherin-mediated adhesion are important for the control of vascular permeability and leukocyte extravasation [36].

## 5. Endothelial Colony-Forming Cells (Ex Vivo Cultured EPC)

The origin and phenotype of blood endothelial outgrowth cells (BEOCs; which are also referred to as endothelial colony formation cells) were first described two decades ago by Lin et al. [22]. To address the origin of BEOC, isolation of samples of mononuclear cells from gender-mismatched bone marrow transplant recipients and seeding to collagen type 1 coated plate was carried out. Consequently, unattached cells were removed after 24 h and only up to 19 endothelial-like cells; 100–200 mononuclear cells were observed to remain on the plate. The latter showed a typical macrophage shape and died out 3 weeks later after plating. Morphologically, BEOCs were observed to have a “cobblestone” shape, highly positive for EPC/EC markers, such as CD34+, CD31 +, VE-cadherin, CD146 +, von Willebrand factor, CD31 +, and negative expression for monocyte marker CD14+, respectively. Notably, upon a fluorescence in situ hybridization (FISH) analysis, the recipient genotype BEOC expansion was gradually decreased in the culture while donor genotypes were dramatically increased by 1023-fold after 1 month, indicating that BOACs were derived from the bone marrow. Fujisawa et al. [37] investigated the origin of EPCs using blood samples from sex-mismatched allogeneic bone marrow transplant patients. The FISH analysis confirmed that BOACs do not originate from the bone marrow, which is controversial to the findings of Lin et al. [38]. Subsequently, Ingram et al. [17] also identified endothelial outgrowth cells in culture between days 14 to 21, termed “endothelial colony-forming cells (ECFCs),” derived from the human peripheral and umbilical cord blood. Therefore, this study utilized a clonogenic method to define the proliferative potential of EPCs at a single-cell level, as hinted by Lin et al. [22]. The in vitro study showed that ECFCs have a high proliferative potential as well as being clonally distinct from CFU-Hill surface antigen expression of ECFCs close to EC while CFU-Hill contaminated with hematopoietic cell subsets [13]. Consequently, several preclinical studies used ECFCs as a therapeutic cell source to cure several life-threatening diseases. The main dilemma in the widespread use of ECFCs is their scarcity in the peripheral blood, equating to 2.5 ECF colonies per milliliter of blood in healthy human subjects, or even using advanced current culture techniques, less than 70% of individuals may form ECFC colonies [27]. A recent paper suggested that depending on the disease state, age, and cell source, the colony formation capability of ECFCs and the numbers are different. For instance, for cord blood vs. children (age 0–10 years), the ECFCs numbers were high (9 and 5), while in the case of middle-aged or aged adults, the level of ECFCs significantly declined up to 0.2 normalized to 10^7^ MNCs [27]. It is presumed that in patients with concomitant diseases, such as diabetes mellites or others, the quantity and quality of EPCs decrease up to zero colony formation [38].

Taken together, since ECFC biology is studied in culture, its in vivo biology and the origin of ECFCs are not defined yet, and further studies are warranted to investigate its origin and in vivo presence.

## 6. EPC Characterization In Vivo

### 6.1. Human EPC Characterization In Vivo

So far, it has been considered a problem in the flow of research that the phenotype and function of EPC had to be discussed in terms of the cultured phenotype “EPCs ex vivo”. From a biological point of view, *the cells that differentiate into vascular endothelial cells in the living body should be defined as EPCs*. However, as is inevitable in biological research development, several discussions have concluded EPC biology based on research studies of cells that differentiate into ECs in culture ex vivo. We need to consider whether these discussions for EPCs ex vivo are ultimately aimed at the cell biology of “EPCs in vivo”. In this regard, we discuss EPC biology based on its origin in vivo. In particular, a majority of initial phase research utilized CD34-positive (CD34+) or CD133-positive (CD133 +) cells in human peripheral blood mononuclear cells and found its commitment into endothelial lineage cell in vitro and its integration into EC in new blood vessel formation in vivo [1,39,40,41,42,43]. These findings were supported by the irrefutable clinical pieces of evidence representing the CD34+-derived cells’ incorporation into the human vasculature. Suzuki et al. reported significant EC replacement by BM-derived cells in acute radiation syndrome patients receiving G-CSF-mobilized CD34+ cell transplantations [44]. The subsequent analysis of the aortas of these patients showed that approximately 25% of all ECs were replaced by ECs originating from donors [44]. Jiang et al. demonstrated that 2% of ECs in the skin and gut from hematologic malignancy recipients who had undergone BM CD34+ cell transplantation were of donor origin [45]. Peters et al. determined that an average of 4.9% donor origin of blood vessels were detected in fluorescence in situ hybridization with sex chromosome-specific probes in the histological samples of various cancers after bone marrow transplantation with donor cells derived from individuals of the opposite sex [46]. Taking together, EPCs are hidden as one of the CD34 cell subsets, and further research is required to address the trace development hierarchy of EPC.

### 6.2. Mouse EPC Characterization In Vivo

The human CD34 (hCD34) cell function is distinct from mouse ones (mCD34). The hCD34+ cells are currently used as a source for hematopoietic transplantation in humans. However, in steady-state murine hematopoiesis, HSCs with long-term reconstitution activity are found almost exclusively in the mCD34-negative to low fraction [47]. Several groups have reported no contribution of transplanted BM-derived cells into the vasculature of implanted tumor grafts in host mice [48]. Others have presented similar negative findings of EPC incorporation into the vasculature and contribution to blood vessel formation using various animal models, including a hindlimb ischemia model [49], a cytokine-induced angiogenesis model [50], a muscle injury model, and a non-injured cerebral nervous system model [51].

In murine hematopoiesis, HSCs are found almost exclusively in the Thy1.1^lo^ lineage (Lin)−/^lo^Sca-1 + population [52]. Within Thy1.1^lo^ Lin−/loSca-1+ cells, HSC activity is enriched in a population expressing receptors for the steel factor c-Kit [47]. It has been demonstrated that bone marrow c-Kit+ / Sca-1 + lineage-negative cells differentiated into two types of EPC colony-forming units (EPC-CFUs), large-sized EPC (large-EPC)-CFUs and small-sized EPC (small-EPC)-CFUs. Gene expression analysis demonstrated that both EPC-CFU-derived cells expressed eNOS, Flk-1, and VE-cadherin, markers of endothelial cells [53]. Transplantation of c-Kit+ / Sca-1 + lineage-negative large-EPCs into ischemic hindlimb enhanced neovascularization in hindlimb ischemia model [53].

In this regard, it is noteworthy to mention that all reports stressing the failure of EPC incorporation and function studies were derived from nonhuman origin, utilizing mouse-based experiments primarily.

## 7. Tissue-Resident EPC

A growing amount of evidence has suggested the existence of tissue-resident EPCs along with circulatory EPCs; additionally, in a lung injury model, tissue-resident pulmonary vascular endothelial cells significantly contributed to vascular repair and also highly expressed CD34, Flk-1/KDR, and c-kit more strongly and fewer CD133 cell surface markers in the proliferative phase rather than the nonproliferating tissue-resident pulmonary vascular endothelial cells in bone marrow chimera mice, indicating that bone marrow-derived lung tissue-resident EPCs mainly contribute to pulmonary vascular repair after the onset of injury [54]. Another elegant EPC fate mapping experiment has clearly shown that tissue-resident pulmonary vascular endothelial cells proliferate in situ on the endothelial layer and that bone marrow-derived EPCs are engrafted into the endothelial layer of lung microvessels at the active barrier repair phase by increasing the number of tissue-resident pulmonary vascular endothelial cell-derived EC (CD45− / CD31+ / BrdU+ / rtTA+) or bone marrow-derived EPC (CD45− / CD31+ / eNOS+ / GFP+) by 22- or 121-fold, respectively [55]. Recently, Wakabayashi et al. identified CD157 as a marker of tissue-resident vascular endothelial stem cells in large arteries and veins of numerous mouse organs [56]. Single CD157 + VESCs form colonies in vitro and generate donor-derived portal veins, sinusoids, and central vein endothelial cells upon transplantation in the liver. In response to the injury, tissue-resident vascular endothelial stem/progenitor cells expand and regenerate entire vasculature structures, supporting the existence of an endothelial hierarchy within the blood vessels [56].

To sum up, the necessity of a new classification of EPC according to their source of origin, phenotype, and function to delineate “true EPC” from “EPC-like” cells is observed. The previous consensus statement on EPCs nomenclature needs to address these questions, as it does not cover all aspects of “EPC” in the literature that have been described so far. A simplified classification may facilitate the appropriate clinical use of these cells.

## 8. Hematopoietic Cells Signal Crosstalk on EPC Development

**Figure 2:**
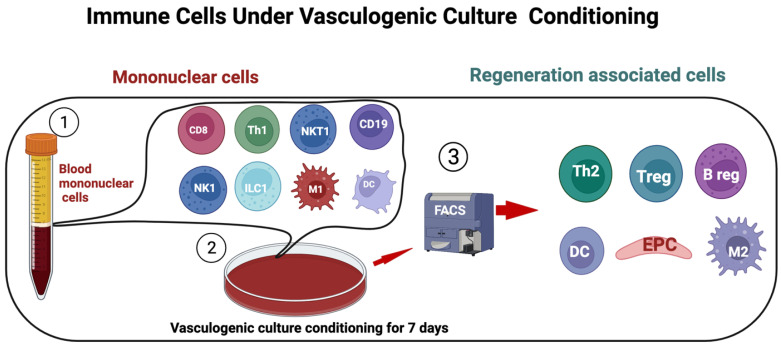
Regeneration-associated cells. PBMCs isolated and cultured in serum-free media with defined growth factor for 7 days and analyzed by flow cytometry. The proinflammatory cell phenotypes shift to anti-inflammatory phenotype cells such as EPC, alternatively activated macrophages M2*ϕ*, Th2, and regulatory T cells.

A seminal EPC study showed that the cocultures of CD34 – (depleted) mononuclear cells with CD34+ cells significantly increased the proliferation rate and tube-like structure formation in fibronectin-coated plates rather than CD34+ cells culturing alone [1]. Subsequentially, studies have also implied that the crosstalk between hematopoietic cells with EPCs plays an important role in terms of EPC differentiation [19]. For example, T cells significantly accelerated primitive or small EPC-CFU, whereas macrophages and megakaryocytes solitarily promoted definitive or large EPC-CFU in in vitro assays in healthy human subjects, indicating that regenerative signals derived from myeloid or lymphoid cell subsets may cause vasculogenic or definitive EPC-CFU maturation [19]. However, over the last two decades, bone marrow mononuclear or purified CD34+ and CD133 + stem/cells transplantation clinical trials have been performed to cure various cardiovascular severe ischemic patients with modest results [57,58,59]. Perhaps, this may couple with qualitative and quantitative analysis, along with crosstalk impairment of EPC with HSC subsets in patients with concomitant diseases, such as diabetes mellites, hypertension, atherosclerosis, and hyperlipidemia, along with risk-associated factors [60,61,62,63,64]. Moreover, the scarcity of EPC in peripheral blood and mobilization obstacles in diabetes mellitus patients hamper the collection of enough cells for therapeutic application in clinical settings [60]. Following considerations, our group has developed a vasculogenic quality- and quantity-controlled ex vivo culture (VC) system to educate mononuclear cells under vasculogenic signaling to increase regenerative cells for tissue regeneration. Notably, under vasculogenic conditioning, naïve PBMCs phenotype converted from proinflammatory (primitive EPC cells, M1*ϕ* monocyte/macrophages (CD192+), Th1 (CD4+ / INF-g+ / IL4−), natural killer cells (CD56+), and B cells (CD19+) etc.) to anti-inflammatory polarized regenerative cells such as definitive or vasculogenic EPC, M2*ϕ* (CD206+), Th2 (CD4+ / INF-g− / IL4+), regulatory T (CD4+ / CD25+ / FoxP3+) and regulatory B cells, and dendritic cells [20,21,65,66] (Figure 2). This educated or regenerative conditioning induced PBMCs to represent as “*regeneration-associated cells*” (RACs) with several important properties of regeneration, such as immune modulatory, anti-inflammatory, and strong vasculogenic effects which were proved in in vivo experiments in different species [21,66,67,68]. The aforementioned characteristics of RACs expand its clinical application ranges, such as cardiovascular ischemic diseases, including peripheral arterial diseases, myocardial impaction, stroke, etc.

The VC technique was applied for peripheral blood-derived mononuclear cells to expand rare EPCs and showed that naïve proinflammatory PBMCs, the latter phenotype, were dramatically shifted to regenerative subsets, such as alternative activated M2, proangiogenic T cells along with definitive or large-sized EPC expansion. In EPC-CFA, vasculogenic conditioned PBMCs dramatically increased large EPC-CFU formation with a high percentage of differentiation, whereas fresh PBMCs formed a few colonies, and most of them were in small EPC-CFU [21,66]. In a hind limb ischemia model of nude mice, we have evaluated RACs therapeutic efficacy vs. control, PBMNC, early EPC, and granulocyte colony-stimulating factor mobilized CD34+ cells-transplanted groups, respectively. The RAC-transplanted groups started to accelerate ischemic leg blood flow perfusion and ischemia tissue recovery in contrast with the compared groups. The underlying mechanism of recovery by RAC may explain that RACs inhibit ischemia-induced inflammatory signals, further leading to reparative process initiation in ischemic tissue, being angiogenesis, arteriogenesis, and myogenesis, while a natural course of ischemia usually includes tissue function loss, such as scar formation [21]. The other merits of RACs and RAC-derived extracellular vesicles are that systemic and small cell dose transplantation can beneficially restore ischemic tissue functions via preferential accumulation at the site of injury. In an acute myocardial ischemia model, we have shown that only a small RACs dose (1 × 10^5^ RACs transplanted via tail vein) markedly augmented left ventricular hemodynamic functions by preferentially homing into the site of injury and developing an additional “biological bypass” to the ischemic host tissues [66,69,70]. Taken together, systemically transplanted RACs easily overcame lung and spleen barriers, preferentially accumulating into injured tissues to set in the motion of blood-derived inflammatory cells phenotype transition into regenerative-associated cells, such as EPCs, M2, Treg, and angiogenic T and B cells in various ischemic organs [21,66,71,72].

## 9. EPC-Derived Extracellular Vesicles

Extracellular vesicles are lipid bilayer particles released from all-nucleated cells and cannot replicate. Depending on the physical size range, EVs divide into small (<100 nm), medium (<200 nm), or large (>200 nm), and usually express CD63 +, CD81 +, Annexin A5, etc. surface markers [73]. Recent studies have demonstrated that EPC-derived extracellular vesicles (EPC-EVs) therapeutic effects are superior to those of its ancestors [69,74,75]. EPC-EVs possess numerous advantages over cell-based therapies in the context of regenerative medicine in terms of (1) cargo delivery of various favorable miRs responsible for angiogenesis, fibrosis, anti-inflammation, and cell proliferation; (2) potential for “off-the-shelf” availability and respective repetitive transplantation; and (3) generally reduced immunogenicity, owing to which allogeneic transplantation is an additional benefit. Previously, we have demonstrated similarities and differences between EPC-derived microRNAs (EPC-miRs) and EPC-EV-derived miRNAs (EPCev-miRs). It can be clearly seen that the majority of EPC-miRs can be found in EPCev-miRs, suggesting a similar transcriptome profile along with the mechanism of action [69]. The mechanism of activation of the angiogenic program in quiescent endothelial cells is linked by the horizontal transfer of genetic materials such as angiomiRs, RNA, and proteins [75]. Perhaps, other beneficial effects work similarly, as we mentioned above. However, angiogenesis-qualified angiomiRs accelerate not only angiogenesis, vasculogenesis, and lymphangiogenesis but also have proliferative, antiapoptotic, and anti-inflammatory effects [69]. Compelling preclinical evidence reveals that EPC-EVs regulate cardioprotection by orchestrating cell angiogenesis, migration and adhesion, cell proliferation, and cell differentiation processes. Mechanistically, cardioprotective properties of EPC-derived EV are associated with miR-218-5p and miR-363-3p overexpression. The latter facilitated cardiac function via enhanced neoangiogenesis and inhibited myocardial fibrosis [76]. Collectively, well-packed EPC-EVs have a great advantage in preserving ischemic tissue from injury, and future studies are warranted to define the beneficial effects of EPC-EVs on other diseases to cure them.

## 10. Unresolved Issues and Future Perspectives

① EPC biomarkers have more questions rather than answers; there are several prospective issues that need to be addressed in this field, such as: Distinct delineating EPC surface markers need to be investigated using state-of-the-art multiomics technology, e.g., single-cell genomics and spatial proteogenomics of whole tissue section analysis to define rare EPCs from circulation and tissue section, but not in the culture (ex vivo and in vivo). Notably, cultured cells are derivatives of circulating cells; however, although their differentiation traits may be ruled out by defined growth factors, the latter may not be corresponded to or apart from real circulating EPCs along with their function.
② It has been believed that EPCs originate from the bone marrow and mobilize in response to ischemia and home to the sites of vascular injury (2, 8). Another earlier study investigated the origins of circulating endothelial cells and endothelial outgrowth from cultures of blood by utilizing FISH of blood samples from bone marrow transplant recipients who had received gender-mismatched transplants. As the data suggest that most circulating endothelial cells in fresh blood originate from vessel walls and have limited growth capability, the outgrowth of endothelial cells from cultures of blood is mostly derived from transplantable marrow-derived cells because these cells have more delayed outgrowth but a greater proliferative rate [22]. However, a recent controversial finding demonstrated that circulating EPCs arise from an alternative niche in the vessel wall but not from the bone marrow by using FISH and was also supported by live-cell imaging, flow cytometry, and immunofluorescence staining methods [37]. Similar findings were revealed by Li et al., wherein the structural integrity of the endothelium of the adult human heart during physiological conditions and following myocardial ischemia is maintained through the clonal proliferation of a subset of resident EPCs with minimal, if any, contribution from bone marrow cells using single-cell technology (58). Another report claimed that the vessel-derived CD34+ cells fraction is crucial for the endothelial repair of injured arteries, but in a mouse model [77]. To define the EPC contribution to vasculogenesis, sex-mismatched, different age, bone marrow transplant patient recruitment and integrated analysis of FISH and multimodal spatial single-cell sequencing technologies to trace the EPCs origin is desired.
③ The cross-talk signals between EPCs and hematopoietic cell subsets, such as monocytes/macrophages, T cells, and B cells, are crucial for EPC differentiation and maintenance, which also need to be paid attention to.
④ All these notable findings need to be involved in EPC classification for the development of an updated consensus since the current consensus on EPC nomenclature has not covered all EPCs [3] by (1) source of origin; (2) and most of the surface molecules to characterizes EPC use are from murine model findings [78], and we strongly discourage from putting these in the same line as a human EPC marker; and (3) in vivo presence, and (4) some misleading information, which we addressed in this manuscript.
⑤ Accumulating evidence has demonstrated that EPC number reduction or biological quality impairment is associated with various diseases. Future large clinical studies warrant establishing the diagnostic and prognostic value of EPC. Moreover, focusing on EPC numbers in peripheral blood and their colony formation capability may be crucial to predicting early cardiovascular adverse events.
⑥ As tissue-resident EPCs gain more attention, modifying them with EPC-derived EVs to boost neovascularization and elderly patients’ EC rejuvenation is desired.

## Data Availability

Not applicable.

## Abbreviations

BOEC	Blood endothelial outgrowth cells
CFA	Colony-forming assay
CFU	Colony-forming unit
EC	Endothelial cells
ECFC	Endothelial colony-forming cells
EPCs	Endothelial progenitor cells
EPC-EVs	EPC-derived extracellular vesicles
FISH	Fluorescence in situ hybridization
hCD34	Human CD34 cells
HECs	Hemogenic endothelial cells
HLI	Hindlimb ischemia
HSCs	Hematopoietic stem cells
HSPCs	Hematopoietic stem and progenitor cells
mCD34	Mouse CD34
PBMCs	Peripheral mononuclear cells
RACs	Regeneration-associated cells
VC	Vasculogenic culture
